# Retrospective Evaluation of GEC-ESTRO Constraints for Definitive Radiochemotherapy with Brachytherapy and Correlation with Oncologic Outcome in Cervical Cancer: A Monocenter Study

**DOI:** 10.3390/cancers16203495

**Published:** 2024-10-15

**Authors:** Tom Schönicke, Raphael Koch, Isabel Vogt, Isabel Falke, Hans Theodor Eich, Gabriele Reinartz

**Affiliations:** 1 Department of Radiation Oncology, University Hospital of Muenster, 48149 Muenster, Germany; isabel.vogt@ukmuenster.de (I.V.); isabel.falke@ukmuenster.de (I.F.); hans.eich@ukmuenster.de (H.T.E.); 2 Institute of Biostatistics and Clinical Research, University of Muenster, 48149 Muenster, Germany; raphael.koch@ukmuenster.de

**Keywords:** radiochemotherapy, cervical cancer, 3D-image-guided brachytherapy (IGBT), Gyn GEC-ESTRO

## Abstract

**Simple Summary:**

Definitive radiochemotherapy represents a well-established and proven treatment option for locally advanced cervical cancer. The Gyn GEC-ESTRO (GGE) provides a protocol for the method and administration of the associated brachytherapy, in which restrictions for target volumes and organs at risk are established through several studies. This study aims to validate the results of the internal treatment protocol and compare them with the GGE recommendations. A sample of 48 patients with locally advanced cervical cancer treated from 2013 to 2023 was retrospectively analyzed. The GGE target volumes were contoured, evaluated, and then compared with patients’ oncologic outcome, as well as bladder and rectal toxicities. The use of MRI during brachytherapy was observed to have a positive effect on survival times in this study. Adherence to the GGE recommendations may also improve local tumor control and time-to-event endpoints, which were comparable to those of other studies. A realistic representation of patient treatment in a radiation oncology center without constant access to MRI for brachytherapy planning was obtained.

**Abstract:**

Background: This study aims to evaluate patients with locally advanced cervical cancer who underwent definitive radiochemotherapy, including brachytherapy, at the University Hospital of Muenster (UKM), focusing on target volume coverage, oncologic outcome parameters, and organs at risk (OAR) toxicities. Results are compared with the Gyn GEC-ESTRO (GGE) recommendations. Methods: Of a cohort of 48 patients, treated between 2013 and 2023, the physical radiation treatment planning with application of CT and MRI and oncologic follow-up data was analyzed. Target volume structures, comprising the high-risk clinical target volume (HR-CTV), intermediate-risk clinical target volume (IR-CTV), Point A, and corresponding EQD2_(α/β=10)_ doses were determined. Endpoints included local tumor control, overall survival (OS), recurrence-free survival (RFS), and progression-free survival (PFS). Total OAR (D2cc) EQD2_(α/β=3)_ doses were correlated with adverse events defined by CTCAE v5.0 and LENT-SOMA criteria. Results: Median follow-up was 58.0 months (95% CI [27.6, 88.4]). FIGO stage I was present in 7 (15%) patients, II in 13 (27%), and III in 28 (58%) patients. A total of 38 (79%) patients showed a complete remission 3 months after treatment. The 5-year event-free rate was 67.4% (95% CI [49.3, 80.3]) for OS, 77.0% (95% CI [56.7, 88.6]) for RFS and 68.1% (95% CI [49.7, 80.9]) for PFS. Incomplete radiation treatment and advanced tumor stages led to worse outcomes. Meeting Point A GGE recommendations increased chances for complete remission and could decrease chances of an event occurring for OS, PFS, and RFS. Compliance with GGE recommendations lowered the chances of OAR toxicity occurring. Conclusions: MRI-based target volume definition for brachytherapy in cervical cancer may improve patients’ OS, PFS, and RFS. Time-to-event endpoints are consistent with comparable studies, and adherence to current ESGO/ESTRO/ESP guidelines is endorsed.

## 1. Introduction

Despite the fact that cervical cancer is preventable, it continues to have a significant impact on women’s health, as it is the fourth most prevalent form of cancer in women globally, in terms of both incidence and mortality [1,2]. Nevertheless, efficacious treatment options exist for cervical cancer and the implementation of screening protocols as prevention is advantageous [2]. Due to the significant impact of cervical cancer, with a given number of 660,000 new cases and 350,000 deaths reported in 2022, the WHO opted to implement a strategy aimed at eradicating cervical cancer, given that detection at early stages can often result in successful treatment [3]. However, guideline-based treatment is often lacking in low- and middle-income countries, and even in high-income countries there are regional limitations [1,4]. For locally advanced cervical cancer (LACC), and thus for patients without distant metastases, definitive radiochemotherapy with curative intent is the standard of care and recommended in the German guideline for the management of cervical cancer [5,6].

In addition to external beam radiotherapy (EBRT), brachytherapy (BT) constitutes an important component of definitive radiochemotherapy for patients with LACC, image-guided brachytherapy (IGBT) being considered the gold standard in this context [2]. For this purpose, the Gynaecology Groupe Européen de Curiethérapie—European Society for Radiotherapy and Oncology (Gyn GEC-ESTRO, GGE) developed current recommendations based on the results of extensive completed and ongoing trials on image-guided intensity modulated external beam radiochemotherapy and MRI-based adaptive brachytherapy for locally advanced cervical cancer (EMBRACE trials) [7,8,9]. Therefore, MRI is of paramount importance in this context, whether used as diagnostic information or for brachytherapy planning. A series of recommendations of the Gyn GEC-ESTRO provide guidelines for the theoretical basis, technical implementation, performance, imaging, and planning of BT [10,11,12,13]. In the course of this development, the EMBRACE-I study reported a 5-year disease-free survival rate of 68% (95% CI [65, 70]) and a 5-year overall survival rate of 74% (95% CI [72, 77]) for 1341 patients [8]. 

Among many others, these oncologic results from EMBRACE-I as exemplary endpoints are the benchmark for modern radiochemotherapy of patients with LACC, particularly since the hypotheses of the EMBRACE-II study aim to exceed these results [7]. Nevertheless, CT imaging remains a prevalent tool in the planning of BT in definitive radiochemotherapy for patients with LACC, partly due to infrastructural challenges [14,15,16]. However, small studies suggest that similar results can be attained through CT-guided BT planning, as demonstrated in a recent study by Sanctis et al. [17]. In a cohort of 67 patients, the 5-year overall survival rate was 76.45% and the 5-year disease-free survival rate was 78.2%, while CT-guided brachytherapy planning was used [17]. 

At the University Hospital of Muenster (UKM), CT-based BT planning is employed as a planning modality alongside the established EBRT and chemotherapy for definitive radiochemotherapy of patients with LACC. The objective of this study is to investigate whether this approach is disadvantageous for patients in terms of organ at risk (OAR) toxicity and oncologic outcomes and how these results compare with those of current studies, including the EMBRACE trials.

## 2. Materials and Methods

### 2.1. Patients and Treatment 

This retrospective, monocenter study obtained ethical approval from the Ethics Committee Westfalen-Lippe (reference 2023-455-f-S). 

A total of 214 patients were treated with cervical neoplasms at the Department of Radiation Oncology of the University Hospital of Muenster (UKM) between 2013 and 2023. Brachytherapy data were available for 56 patients who received definitive radiochemotherapy, of which 48 patients met the inclusion criteria. These criteria were defined as the confirmed diagnosis of locally advanced, invasive cervical cancer without distant metastases. All patients were to receive a definitive radiochemotherapy with image-guided brachytherapy (IGBT) as treatment with a curative approach.

**Figure 1:**
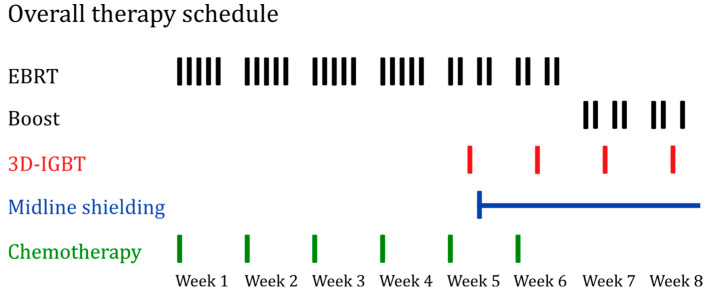
Overall therapy schedule, with each line representing a fraction. EBRT (black) with 28 fractions of 1.8 Gy, totaling 50.4 Gy. A potential boost could extend the dose to 63 Gy. Midline shielding (blue) starts at 39.6 Gy. Four fractions of IGBT (red) are administered (under midline shielding EBRT) with 6 Gy each, for a total dose of 24 Gy. No EBRT fractions were applied on the days of brachytherapy. Chemotherapy (green) was administered 5 to 6 times in total, once per week, with a cisplatin dose of 40 mg/m^2^ BSA. Abbreviations: EBRT, external beam radiotherapy; 3D-IGBT, 3D-image guided brachytherapy.

The target volume, including uterus, cervix, the parametria and tumor-infiltrated parts of the vagina, was initially irradiated to 39.6 Gy via external beam radiotherapy (EBRT). A native computed tomography (CT) scan provided the imaging basis for radiation planning. If a diagnostic magnetic resonance imaging (MRI) was performed prior to the entire therapy, it was also used to determine the extent of the tumor. Once brachytherapy started, the EBRT was continued with midline shielding, delivering a total dose of up to 50.4 Gy. In the event of parametrial infiltration, a further boost of up to 55.8 Gy via EBRT was administered. Additionally, the parametria and individual enlarged lymph nodes could be boosted up to 63 Gy via EBRT. The irradiation was delivered with a single dose of 1.8 Gy in five fractions per week using intensity-modulated radiation therapy (IMRT) and image-guided radiotherapy (IGRT). Treatment lasted approximately 6 to 8 weeks, depending on the selected therapy schedule for the corresponding and individual patient situation, further elaborated in Figure 1.

An intracervical sleeve was placed shortly before the first brachytherapy was performed. The 3D-IGBT starting from 39.6 Gy was physically planned predominantly based on a native CT scan with the brachytherapy applicator in place. The target volume covered the individual tumor region. Vaginal dose administration was applied to a tissue depth of 5 mm. The cervical dosage was oriented to Point A with a tissue depth of 2 cm, however, it was also individually adapted in 3D planning to the respective tumor extension and infiltration depth. A total of four fractions of high-dose-rate (HDR) brachytherapy with a single dose of 6 Gy were given once a week using the afterloading technique. The standard prescribed dose was identical for all patients, the irradiated volumes were individually adjusted taking into account the corresponding OAR dose exposure and tolerance. The UKM internal constraints per brachytherapy application for the organs at risk (OAR) are derived from the constraints defined in a publication by Shaw et al. and mathematically converted into the fractionation scheme of 6 Gy applied 4 times by the UKM [18]. This derivation results in UKM internal OAR constraints per fraction of <6.23 Gy (D2cc) for the bladder and <4.26 Gy (D2cc) for the rectum. 

MRI was performed prior to EBRT or for brachytherapy depending on individual patient circumstances (tumor extension, anatomical situation) and infrastructural availability. The MRI imaging was predominantly performed without the brachytherapy applicator in place to assess the tumor situation and for fusion with the planning CT. If an MRI was performed in addition to the CT for brachytherapy application, a maximum interval of 4 days between the MRI and the application was tolerated for MRI-imaging. For the patients who received an MRI for brachytherapy application, image-guided brachytherapy was planned in the majority of cases on the basis of MRI-CT fusion. In these cases, the T2-weighted image was employed in accordance with the Gyn GEC-ESTRO (GGE) guidelines [12]. Apart from that, a sole MRI-based physical planning of brachytherapy was used in three individual cases with challenging anatomical conditions. In these cases, the brachytherapy applicator was included in the MRI imaging. Despite the availability and inclusion of T2-weighted images, technical constraints necessitated the additional use of T1-weighted images for these MRI-based plan calculations only. If a positron emission tomography (PET)-CT was performed, the CT component was treated exclusively as a diagnostic CT scan, without usage of any information derived from the PET component. If no CT was available during brachytherapy, the plan of the previous fraction was used, which was only necessary once for technical reasons.

Chemotherapy was typically administered with cisplatin at 40 mg/m^2^ body surface area (BSA) weekly for at least five cycles. One patient received concurrent chemotherapy with mitomycin C (10 mg/m^2^ BSA) in week 1 and 5 instead of cisplatin. Figure 1 further describes the overall therapy schedule for EBRT with a potential boost, 3D-IGBT, and chemotherapy in detail. It was developed in alignment with the representation of treatment schemas in the EMBRACE-II study protocol [19].

### 2.2. Technical Details

The main brachytherapy device used in 47 patients from 2013 to 2023 was the MicroSelectron HDR V3 by Elekta (Stockholm, Sweden). Only one patient received brachytherapy via the Gammamed 12i from Sauerwein, currently known as Varian (Palo Alto, CA, USA), in 2013. In all patients, a vaginal cylinder CT/MR compatible (multichannel) applicator in combination with an angled intrauterine applicator was used. The angle of the intrauterine component of the applicator was set individually for each patient with variants including 15, 30, and 45 degrees. The length of the smit sleeve used for CT/MRI was 40 mm. The radionuclide used was Ir-192, with a maximum single activity of 444 GBq. For brachytherapy, Oncentra Brachy Ver. 4.6.3 by Elekta served as the planning system. Eclipse Ver. 15.6 by Varian Medical Systems, Inc. was used as the treatment planning system for EBRT, which was performed on the linear accelerator TrueBeam from Varian.

### 2.3. Recorded Data 

Assessed data covered the following parameters: age at the initiation of therapy, pathological subtype, administration of chemotherapy, and FIGO stage standardized according to the 2018 classification [5]. 

Target volumes, including residual gross tumor volume (GTVres), high-risk clinical target volume (HR-CTV), intermediate-risk clinical target volume (IR-CTV), and Point A, were retrospectively contoured into the radiation plans in accordance with GGE recommendations [11]. The accuracy of MRI-CT imaging fusion and the accurate delineation were reviewed by two radiation oncologists. Point A was defined as a volume of 0.5 × 0.3 × 0.3 cm due to technical reasons (facilitating data extraction, with the extracted dose being equivalent to a point dose). The mean dose of this volume was used for evaluation as the required ‘Point A dose’. Additionally, the initial tumor volume before therapy (GTVpri) was recorded.

Doses were defined as D98 for GTVres, HR-CTV, IR-CTV, and additionally as D90 for HR-CTV, based on GGE and EMBRACE-II planning aims [7,10]. Volume data, including V150 and V200 for HR-CTV and IR-CTV, were also acquired. Volumes for GTVres, and HR- and IR-CTV presented at the last brachytherapy application were chosen. The specified dose for V150 and V200 was defined as the respective single dose of brachytherapy, meaning 6 Gy in most cases. The average percentage for the V150 and V200 values of all four brachytherapy applications of each patient was therefore calculated and used for the analysis.

For organs at risk (OAR), bladder and rectum were considered and D2cc was collected as per GGE recommendation [10]. Vaginal and dermal complications were also listed. Acute complications were evaluated using the Common Terminology Criteria for Adverse Events (CTCAE) v5.0, while long-term complications were assessed using the Late Effects in Normal Tissues—Subjective, Objective, Management, Analytic (LENT-SOMA) scale [20,21]. Acute complications were defined as those occurring during therapy or within 30 days afterwards. Long-term complications were cumulatively recorded as they occurred during the follow-up period.

Total doses result from an equivalent dose at 2 Gy fractions (EQD2) for brachytherapy combined with EBRT. For brachytherapy, the total EQD2 dose was calculated from all four fractions, while for EBRT, the EQD2 dose was determined from a composite plan. For EQD2 calculations, the formulas $BED=nd\left(1+\frac{d}{\alpha /\beta }\right)$ and $EQD2=\frac{BED}{1+2/\alpha /\beta }$ were used, where n equals the number of fractions and d represents the dose per fraction [10]. The α/β ratio was set as 3 for normal tissues and 10 for the tumor [10]. 

Ultimately, dose deviations from EMBRACE-II planning targets were calculated, and the patients were grouped accordingly. If specified in EMBRACE-II, the hard constraints were used; otherwise, the soft constraints were applied [7]. Dose deviations were calculated by subtracting the administered dose from the GGE recommendation.

Furthermore, overall survival (OS), progression-free survival (PFS), and recurrence-free survival (RFS) were documented. PFS was defined as the time from the end of therapy to either the progression of the disease or the date of the patient’s death, whereby only patients with at least a stable disease were included. RFS was defined as the months from achieving a complete remission to disease recurrence or death, with only patients with a complete remission included. Overall survival was defined as the time elapsed between end of treatment and the occurrence of either death or the last follow-up. For all survival times, patients without an event were censored at their last follow-up. Local tumor control was assessed three months after the completion of therapy via current imaging and gynecological examination results. It was differentiated between complete remission, partial remission, stable disease, and progressive disease.

### 2.4. Statistical Analysis

Statistical analyses were performed using IBM SPSS Statistics for Windows, Version 29 (IBM, Armonk, NY, USA). The analysis of time-to-event outcomes was conducted using log-rank tests as well as Cox proportional hazard regression models. The event-free rates at three and five years were reported as Kaplan–Meier estimates and pointwise 95% confidence intervals (CI), calculated using the complimentary log–log transformation. The median follow-up time including the 25% to 75% quantiles (Q25–Q75) were estimated using the reverse Kaplan–Meier method. To determine the univariable influence on OS, PFS, and RFS, univariable Cox regressions were performed using total doses of the target volumes, deviations from GGE recommendations, completion of therapy, age, presence of MRI, volume measurements including V150, V200, and tumor stage including lymph node status. The results are presented as hazard ratios (HR) with corresponding 95% confidence intervals. Differences in time-to-event endpoints regarding presence of MRIs, lymph node involvement, completion of therapy, FIGO stage, and compliance with Point A recommendations were investigated using log-rank tests. Associated Kaplan–Meier curves with 95% CI using log–log transformation were created using the statistical software R, version 4.4.1 [22].

Binary logistic regressions were applied to examine the univariable influences on local tumor control (complete remission yes vs. no). Results are reported as odds ratios and 95% confidence intervals. Differences for deviations of GGE recommendations and presence of MRI were analyzed via Mann–Whitney U test. For correlation between volume of different target volumes and deviations from GGE recommendations, as well as between initial tumor volume and its percentage as well as absolute reduction over the course of therapy, scatterplots were investigated and Spearman’s rank correlation coefficients including 95% confidence intervals were calculated. Regarding OAR toxicities, logistic regressions were applied to determine the influence of D2cc and compliance with GGE recommendations on the binary classification of occurred complication of any grade versus no complication. Results are reported as odds ratios and 95% confidence intervals.

All *p*-values and confidence limits were two-sided and were aimed to be exploratory in nature, instead of confirmatory. Consequently, no adjustments for multiplicity were made. If an exploratory two-sided *p*-value was below 0.05, it was considered statistically noticeable.

## 3. Results

In the following, Table 1, Table 2, Table 3 and Table 4 show the results for the influences on time-to-event endpoints, specifically PFS, OS, and RFS, which were analyzed through the application of both log-rank tests and Cox proportional hazard regression models. The output of the logistic regression model used for the binarily operationalized local tumor control is also displayed.

### 3.1. Patients Characteristics and Follow-Up 

Out of the 48 patients investigated, the median age at the start of treatment was 50.5 years (range 27 to 77). Regarding histopathological classification, squamous cell carcinoma was present in 40 cases (83%), while adenocarcinoma was present in 8 cases (17%). According to the FIGO 2018 classification of cervical cancer, stage IB was diagnosed in 2 patients, IB1 in 3, IB2 in 1, IB3 in 1, IIA2 in 1, IIB in 12 (25%), IIIB in 4, IIIC1 in 11 (23%), and IIIC2 in 13 (27%) patients. Consequently, 24 (50%) patients had no lymph node involvement, 11 (23%) patients had pelvic, and 13 (27%) had para-aortic lymph node metastasis. 

Out of the 48 patients, 38 (79%) were in complete remission (CR) 3 months after therapy ended. Three patients were in partial remission, two in stable disease and five (10%) were in progressive disease. A total of 48 patients were evaluated for overall survival. Altogether, 43 (90%) patients with at least stable disease 3 months after therapy were included for progression-free survival and 38 (79%) patients with complete remission were analyzed for recurrence-free survival. The median duration of the entire treatment was 22 days (IQR 21–28.5) with a minimum of 14 and a maximum of 60 days.

For OS, the estimated survival probability was 71.9% (95% CI [55.4, 83.2]) at 3 years and 67.4% (95% CI [49.3, 80.3]) at 5 years. PFS at 3 years was 76% (95% CI [59.9, 86.3]), and at 5 years it was 68.1% (95% CI [49.7, 80.9]). RFS was 85.8% (95% CI [69.0, 93.8]) at 3 years and 77.0% (95% CI [56.7, 88.6]) at 5 years. Cause of death was the tumor condition in 13 out of 14 cases, with the cause remaining unknown in one case. Estimated median follow-up was 58.0 months (95% CI [27.6, 88.4], (IQR 84–28).

As for age at the start of therapy, older age was associated with an increased risk of death during the observation period (HR = 1.05, 95% CI [1.01, 1.09], *p* = 0.027). Concerning tumor stage, if differentiated between FIGO stage I, II, and III, and FIGO I being considered as the reference category, we observed that with increasing tumor stage, the risk of experiencing an event related to progression-free survival, recurrence-free survival, and overall survival increases, except for FIGO stage II regarding RFS, as listed in Table 2, Table 3 and Table 4.

**Figure 2:**
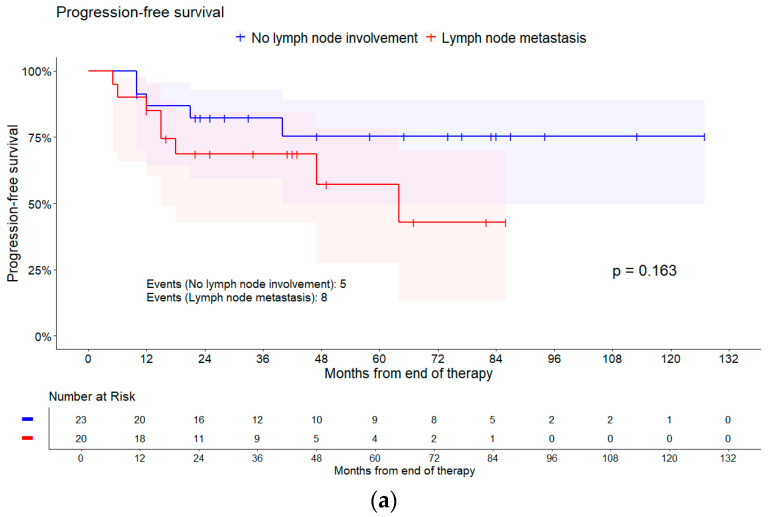

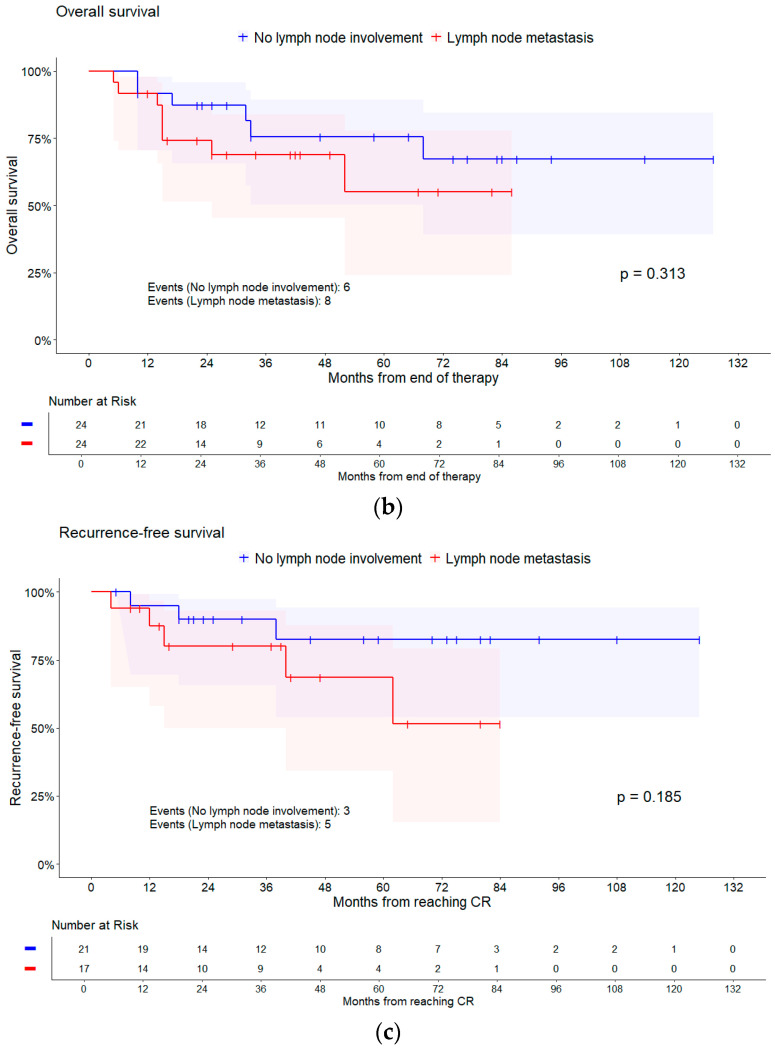
Kaplan–Meier curves of different survival times for lymph node involvement. Shaded areas represent pointwise 95% CI (log–log transformation). *p*-values are from log-rank tests. (**a**) Progression-free survival; (**b**) overall survival; and (**c**) recurrence-free survival.

About lymph node status, the chance for having a complete remission decreased with pelvic lymph node involvement (OR = 0.38, 95% CI [0.06, 2.29], *p* = 0.292) and para-aortic lymph node metastasis (OR = 0.32, 95% CI [0.06, 1.74], *p* = 0.188) compared to no lymph node involvement. Furthermore, as displayed in Table 2, especially patients with para-aortic lymph node metastasis have an increased risk of experiencing an event related to PFS compared to those with no lymph node involvement (HR = 3.04, 95% CI [0.86, 10.76], *p* = 0.192). In addition, patients with para-aortic lymph node metastasis had a worse PFS compared to patients with pelvic lymph node metastasis (HR = 2.48, 95% CI [0.78, 7.92], *p* = 0.192). This is displayed in the Kaplan–Meier plots with the log-rank test in Figure 2.

### 3.2. Treatment 

#### 3.2.1. Imaging and Completion of Therapy

Among the 48 patients, 33 (69%) received a complete therapy. Among the 15 (31%) patients with incomplete therapy, 4 received only incomplete brachytherapy, 10 (21%) received only incomplete chemotherapy, and 1 had both incomplete brachy- and chemotherapy. Reasons for incomplete brachytherapy included issues with tolerability and excessive doses to the organs at risk (OAR). Reasons for receiving fewer chemotherapy cycles included hematological limitations such as thrombocytopenia and leukopenia. EBRT was completed in every case examined. A total of 34 (71%) patients received an EBRT boost, while a boost for the remaining 14 (29%) patients was not indicated.

Regarding the MRIs performed before and during therapy, 14 (29%) patients did not receive an MRI, while for 34 (71%) patients, at least one MRI was performed before EBRT, during brachytherapy, or both. With exclusive focus on brachytherapy, 32 (67%) patients did not have an MRI available for any brachytherapy application, while 16 (33%) patients had at least one MRI available for individual 3D planning or as a basis for target volume definition.

**Figure 3:**
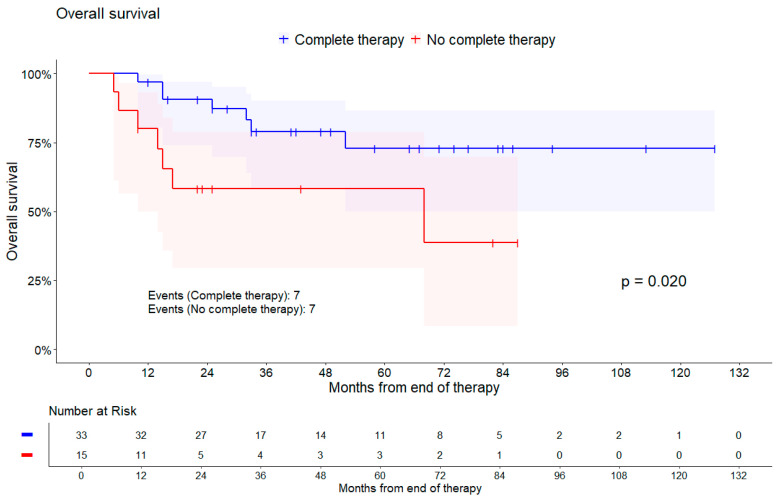
Kaplan–Meier curve of overall survival comparing patients with complete therapy against those without. Shaded areas represent pointwise 95% CI (log–log transformation). *p*-value is from log-rank test.

Patients with incomplete therapy had a higher risk of death (HR = 3.27, 95% CI [1.13, 9.42], *p* = 0.020). Figure 3 represents the Kaplan–Meier plot for this outcome. Incomplete therapy was also associated with lower RFS, (HR = 2.16, 95% CI [0.51, 9.11], *p* = 0.284), but not with PFS (HR = 1.07, 95% CI [0.29, 3.91], *p* = 0.921). Additionally, the chance for having a complete remission decreased with no complete therapy (OR = 0.61, 95% CI [0.14, 2.60], *p* = 0.505). A tendency towards a higher chance of achieving a complete remission was observed in patients with an additional EBRT boost (OR = 1.87, 95% CI [0.44, 8.01], *p* = 0.401).

**Figure 4:**
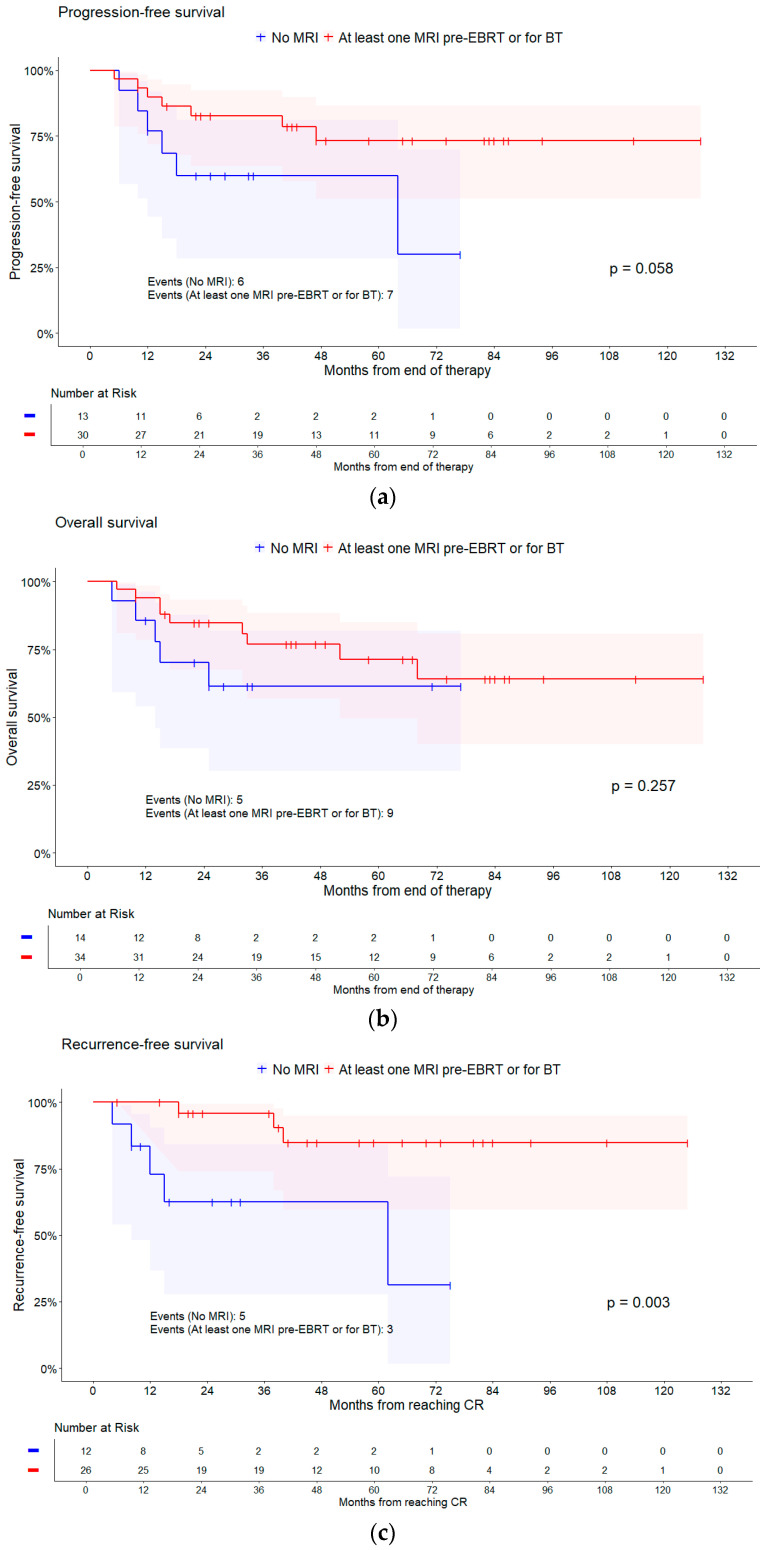

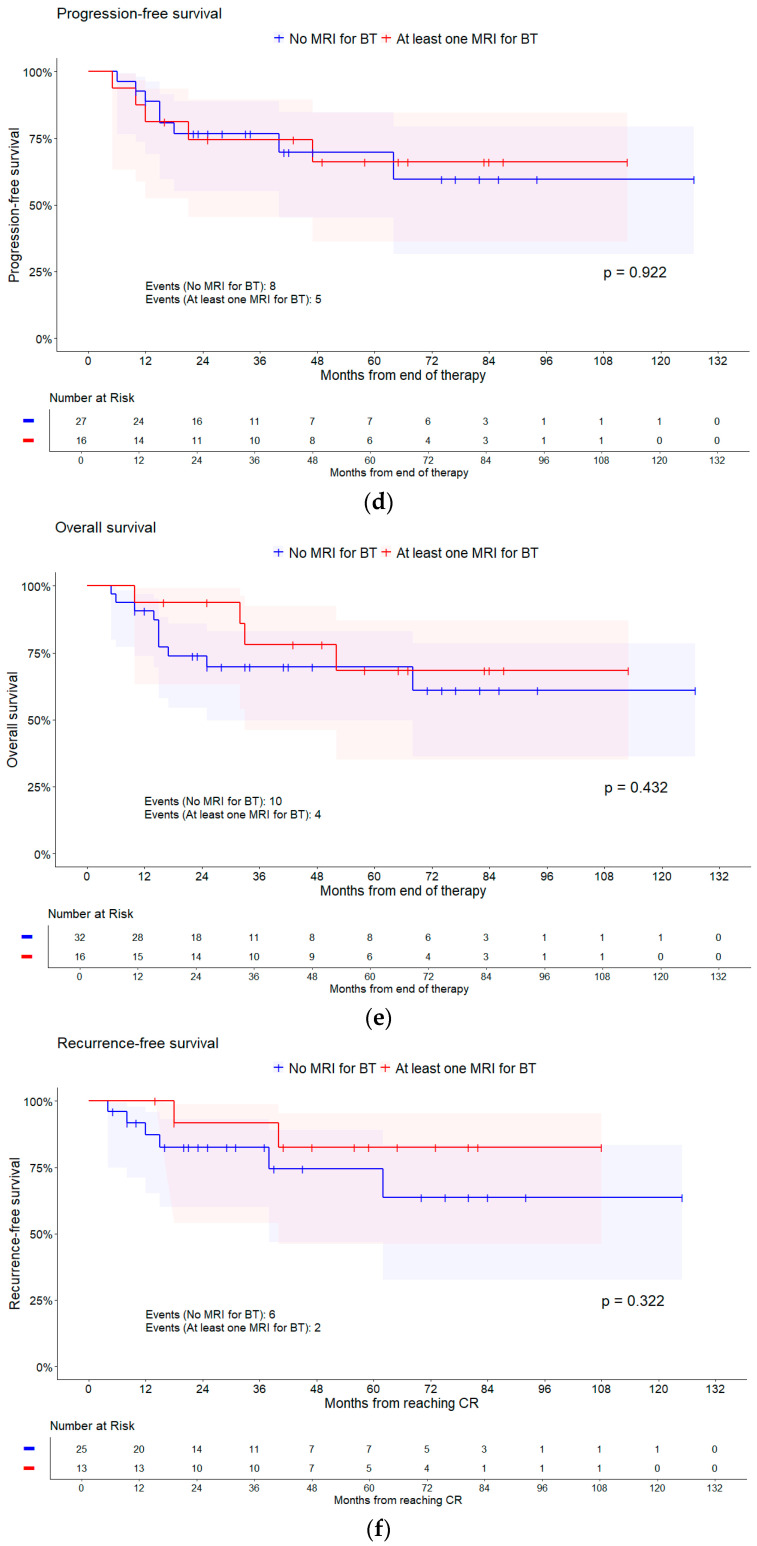
Kaplan–Meier curves of different survival times for having any MRI and for having an MRI for brachytherapy (BT). Shaded areas represent pointwise 95% CI (log–log transformation). *p*-values are from log-rank tests. (**a**) PFS for MRI; (**b**) OS for MRI; (**c**) RFS for MRI; (**d**) PFS for MRI BT; (**e**) OS for MRI BT; and (**f**) RFS for MRI BT.

Focusing on MRI scans performed, the hazard of an event tended to be lower for PFS (HR = 0.36, 95% CI [0.12, 1.09], *p* = 0.058), RFS (HR = 0.14, 95% CI [0.03, 0.61], *p* = 0.003), and OS (HR = 0.54, 95% CI [0.18, 1.62], *p* = 0.257), i.e., all survival times tended to be better, if an MRI was performed. The same trend shows when differentiating between no or at least one MRI for any brachytherapy application for OS and RFS, as shown in Table 3 and Table 4. No influence on PFS regarding the performance on MRI scans could be observed, as results in Table 2 indicate. Kaplan–Meier curves are shown in Figure 4.

#### 3.2.2. Dose–Volume Parameters

Table 5 represents the descriptive analysis of the total doses for the dose–volume parameters of the GTVres, HR-, and IR-CTV for all 48 patients. All total doses discussed for different target volumes are treated as an EQD2_(α/β=10)_. The deviations from Gyn GEC-ESTRO recommendations for HR- and IR-CTV, which are based on EMBRACE-II planning aims, are listed in Table 6 [7]. Dose deviations are presented as continuous variables, because no reviewed patient met the recommendations. Concerning Point A, however, 21 (44%) patients met the recommendations. The median of the Point A dose EQD2_(α/β=10)_ measured 66.1 (IQR 62.3–69.9) Gy, with a minimum of 51.7 Gy and a maximum of 83.6 Gy.

Regarding volumes, the median volume of the GTVres was 18.5 cm^3^, with a minimum of 2.2 cm^3^ and a maximum of 70.2 cm^3^. For HR-CTV, the median volume was 44.2 cm^3^, with a minimum of 7.2 cm^3^ and a maximum of 111.4 cm^3^. The IR-CTV volume had 141.45 cm^3^ as the median, with a minimum of 50.2 cm^3^ and a maximum of 260 cm^3^.

When analyzing the relationship between total doses and survival times, no noticeable influences were found. When analyzing local tumor control, no influence of total doses on the chance of having a complete remission could be shown. Still, Table 1 indicates a slight trend towards a higher chance of having a complete remission per increasing unit of the total doses to target volumes.

Additionally, there was a higher chance of complete remission when V150 (OR = 1.30, 95% CI [0.98, 1.73], *p* = 0.073) or V200 (OR = 1.77, 95% CI [1.01, 3.09], *p* = 0.046) of the IR-CTV increased. Comparable outcomes presented for increasing V150 (OR = 1.12, 95% CI [0.99, 1.27], *p* = 0.063) and V200 (OR = 1.22, 95% CI [0.98, 1.50], *p* = 0.073) of the HR-CTV. This suggests that with increasing V150 and V200 of the HR- and IR-CTV, local tumor control could improve.

Discussing the deviations from GGE recommendations, no influence on survival times and risks of events could be observed. For local tumor control, there was no influence of deviations from GGE on the chance of achieving complete remission. However, as Table 1 indicates, there was a slight trend that local tumor control decreases with increasing deviations from the GGE recommendations. Nevertheless, no differences in deviation from GGE recommendations based on MRI presence could be observed (Mann–Whitney U test, all *p* > 0.05). 

Meeting the Point A GGE recommendation could increase the chance of having a complete remission regarding local tumor control (OR = 1.38, 95% CI [0.34, 5.56], *p* = 0.655). Meeting the recommendations could furthermore lower the chance of an event occurring regarding PFS (HR = 0.62, 95% CI [0.21, 1.86], *p* = 0.385), RFS (HR = 0.72, 95% CI [0.18, 2.86], *p* = 0.631), and OS (HR = 0.88, 95% CI [0.31, 2.51], *p* = 0.806).

If the volume of different target volumes is taken into consideration, scatterplots indicate a positive linear correlation between the volume of GTVres and HR-CTV and the corresponding deviations from GGE recommendations of the GTVres D98 and HR-CTV D90 and D98. A small positive correlation between GTVres volume and D98 deviation was observed (Spearman’s *ρ* = 0.38, (95% CI [0.10, 0.60])) and additionally between HR-CTV volume and D90 (Spearman’s *ρ* = 0.37, (95% CI [0.09, 0.60])) and D98 deviation (Spearman’s *ρ* = 0.25, (95% CI [−0.05, 0.51])). 

Furthermore, when taking the percentual and absolute difference between initial tumor volume and volume of GTVres at the last brachytherapy into consideration and comparing it with initial tumor volume, the scatterplot reveals a positive monotone association. There is a monotone correlation between the initial tumor volume and the percentage reduction in volume (Spearman’s *ρ* = 0.52, (95% CI [0.27, 0.70])) as well as absolute reduction (Spearman’s *ρ* = 0.91, (95% CI [0.83, 0.95])). With a larger initial tumor volume, the tumor had a higher relative and absolute volume reduction over the course of therapy.

### 3.3. Organs at Risk 

Bladder and rectum were the OAR of primary interest. Median D2cc EQD2_(α/β=3)_ for bladder was 79.0 Gy (interquartile range 71.5–85.4) with a maximum of 105.3 Gy and a minimum of 58.6 Gy. The median rectum D2cc was 73.5 Gy (interquartile range 65.9–78.5) with the maximum at 98.7 Gy and minimum at 49.0 Gy. The soft constraint for the bladder regarding GGE recommendations was met in 25 (52%) cases. The hard constraint was fulfilled in 18 (38%) cases and 5 (10%) patients were outside the recommendations. For the rectum, the soft constraint was fulfilled in 10 (21%) cases, the hard constraint in 19 (40%) and 19 (40%) of the patients did not meet the recommended D2cc doses. 

The individual doses of each brachytherapy application were evaluated using a linear mixed model with a random intercept for the patient. The estimated mean dose for D2cc of the total 189 brachytherapy applications for the bladder was 4.79 (95% CI [4.64, 4.94]) Gy (range 2.0–7.0). The corresponding estimated mean D2cc for the rectum was 4.71 (95% CI [4.54, 4.88]) Gy (range 1.6–6.2). In 187 (99%) applications, the UKM internal constraints for the bladder in brachytherapy application were met. For the rectum, the number drops to 37 (20%) applications, where the constraints were fulfilled.

Table 7 demonstrates the different complication frequencies for bladder and rectum. Regarding acute vaginal or dermal complications, 34 (71%) patients had no complications. Eleven (23%) patients had grade I and three patients had grade II complications. Concerning long-term complications, only 43 of 48 patients could be evaluated due to missing retrospective data, 16 (33%) patients had no complications recorded, for grade I there were 5 (10%) patients documented, 11 (23%) for grade II, 9 (19%) for grade III, and 2 patients had grade IV complications. 

To distinguish between any complication regardless of grade and no complication, a logistic regression model was used. Regarding acute complications, the likelihood of developing an acute bladder complication tended to be lower in patients fulfilling the hard constraint of the GGE recommendations (OR = 0.53, 95% CI [0.07, 4.01], *p* = 0.541) and in patients fulfilling the soft constraint (OR = 0.31, 95% CI [0.04, 2.27], *p* = 0.250) compared to patients who missed any constraint, respectively. Similar results were observed for the rectum and acute complications, where adhering to the hard constraint (OR = 0.77, 95% CI [0.19, 3.16], *p* = 0.721) and the soft constraint (OR = 0.24, 95% CI [0.05, 1.21], *p* = 0.084) tended to lower the chances of any acute complication occurring compared to those missing any constraint. Odds ratios could therefore indicate that adhering to the constraints established by the GGE decreases the chance of acute complications of any grade occurring. 

D2cc did not affect the likelihood of any acute complications occurring for the bladder. Concerning the rectum per increasing unit of D2cc, the chance of experiencing any acute complications could similarly increase (OR = 1.05, 95% CI [0.98, 1.13], *p* = 0.151). For the chance of developing long-term complications, regardless of grade, no decisive influences of D2cc or compliance with GGE recommendations on the bladder could be observed. Nonetheless, increasing D2cc could elevate the odds of experiencing long-term complications for the rectum (OR = 1.03, 95% CI [0.96, 1.11], *p* = 0.449). There was no evident influence of GGE deviations on long-term rectum complications. 

Analyzing vaginal and dermal complications, a slightly increased chance of any long-term complication occurring per increasing unit D2cc of the rectum could be observed (OR = 1.06, 95% CI [0.98, 1.15], *p* = 0.160). Furthermore, adhering to the rectum hard constraint (OR = 0.46, 95% CI [0.11, 1.96], *p* = 0.292) and soft constraint (OR = 0.33, 95% CI [0.06, 2.00], *p* = 0.229) might also lower the chance of developing long-term vaginal and dermal complications in comparison with patients fully outside the recommendations. 

## 4. Discussion

The objective of this study was to evaluate the Gyn GEC-ESTRO recommendations and constraints for brachytherapy in 48 patients with locally advanced cervical cancer who were treated in the Department of Radiation Oncology, University Hospital of Muenster (UKM), Germany. In particular, the study will investigate how the requirements of the GGE guidelines can be transferred to the therapeutic approach at this center, whether an additional diagnostic MRI during brachytherapy or before EBRT plays a central role in this context, and what possible effects this may have on endpoints such as local tumor control and different survival times. The Gyn GEC-ESTRO guidelines were correlated with patients’ oncologic outcomes and OAR complications through a retrospective integration of the GGE constraints into the radiation plans.

The Gyn GEC-ESTRO exerted a profound impact on the development of radiotherapy for cervical cancer. From EMBRACE-I, which provided prospective multicenter data for analyzing GGE recommendations, to EMBRACE-II, which interprets and extends the findings from EMBRACE-I and RetroEMBRACE, among others, substantial progress was made [7,8,23,24]. Comparing brachytherapy as a boost modality in the treatment of cervical cancer reveals its superiority over other modalities, especially the stereotactic body radiotherapy (SBRT) boost, particularly in terms of progression-free survival, overall survival, and the incidence of toxicity [25,26]. The application of magnetic resonance imaging-based image-guided adaptive brachytherapy (IGABT) is demonstrably superior to conventional brachytherapy (CBT) and implements MRI as the gold standard. These treatment protocols were demonstrated to achieve good local tumor control while simultaneously minimizing toxicity [27]. Nevertheless, universally implementing MRI for brachytherapy planning remains a major challenge, in part due to infrastructural limitations [15,16]. In a recent review, despite MRI being the benchmark, CT-based planning for brachytherapy was the most commonly used method [14]. As for this study, usage of MRI was rather limited, with only 16 (33%) patients having at least one MRI available for brachytherapy. Older and more recent investigations indicate that the usage of CT-guided brachytherapy results in inferior contouring outcomes, with an overestimation of tumor size in comparison to MRI imaging [15,28]. Comparison of the HR-CTV volumes, with a median of 44.2 cm^3^ (IQR 29.5–63.1), with those reported in EMBRACE-I, which reported a median of 28 cm^3^ (IQR 20–40), suggests that this effect may have influenced target volume contouring. Furthermore, the D2cc values of organs at risk may also be adversely affected when CT is used for brachytherapy planning in contrast to MRI [29]. The GEC-ESTRO therefore developed a solution for CT use [30]. Considering the contouring physicians at this center and the inter-observer variation in delineation for brachytherapy based on CT planning, a study indicated good concordance between the contours despite different levels of expertise [31]. Nevertheless, a review of target volumes and OAR delineation should be performed by experienced physicians, which was carried out consistently within the study. Since the results of this study demonstrate that the use of MRI can potentially improve patients’ oncologic outcomes regarding time-to-event endpoints, the MRI-based target volume definition for brachytherapy should be recommended.

In order to conduct a meaningful assessment of patients’ oncologic outcomes and OAR toxicities, a comparison with studies that also carried out CT-based brachytherapy planning on a similar patient cohort and follow-up period is essential. Two recent studies by Xiu et al. (2022) with 211 patients and a median follow-up of 69 months and Chan et al. (2022) with 135 patients and a median follow-up of 54 months, while not fully comparable, offer indicative points of comparison with this research [32,33]. At 5 years, disease-free survival was 67% for Xiu et al., and for Chan et al. 65.2% [32,33]. Progression-free survival rate in this present study reached a resembling 68.1% (95% CI [49.7, 80.9]) at 5 years. With regard to the overall survival rate at 5 years, while Xiu et al. reported 78%, Chan et al. demonstrated an overall survival rate of 87.2%, respectively [32,33]. In this study, overall survival at 5 years was 67.4% (95% CI [49.3, 80.3]). The previously shown findings indicate that lymph node involvement, particularly with para-aortic metastasis, may lead to poorer local tumor control and increased risk of events within the survival times. The somewhat inferior overall survival rate observed in this study could be attributed to the fact that only 2.2% of patients in Chan et al. had para-aortic lymph node involvement, compared to 27% in this study [33]. Moreover, for Chan et al., the most prevalent FIGO stage according to the 2009 classification was IIB, with a frequency of 45.9% among patients. In contrast, the most common FIGO stage in this study was IIIC, occurring in 50% of cases [33]. It should be noted that different FIGO classifications were used [33]. The study by Xiu et al. predominantly recorded the FIGO stages IIIB with 44.1% and IIB with 42.2%, which also reflects a substantial lower incidence of para-aortic lymph node involvement and notably less advanced tumor stages [32]. However, with regard to the coverage of retrospectively added target volumes, the median D90 EQD2_(α/β=10)_ of the HR-CTV is relatively low at 55.0 Gy (IQR 51.8–58.6), in comparison to 84 Gy (±7.54) from Chan et al. and 91.0 Gy (±6.0) from Xiu et al. [32,33]. In general, the time-to-event endpoints are comparable to those observed in these two partially similar studies.

It is already well established that sparing organs at risk is effectively achieved in brachytherapy planning by means of application of MRI imaging [34]. With the use of CT, however, comparing the incidence of toxicities in this study with those of Xiu et al., despite the longer yet approximate follow-up time, gastrointestinal and rectal adverse events of a high grade (≥G3) occurred with similar frequency of cumulative 12.3% from Xiu et al., which are nearly identical to the 12.5% reported in this study [32]. No cases of high-grade bladder toxicity were recorded by Xiu et al. [32]. In this study, 11 (23%) patients presented high-grade long-term bladder toxicity, with 9 experiencing high-grade incontinence and 2 developing fistulas. It should be noted that, unlike Xiu et al., this study applied the LENT-SOMA criteria instead of the EORTC-RTOG criteria [32]. In accordance with the LENT-SOMA criteria, high-grade incontinence is defined as the regular use of multiple pads per day [21]. It is important to consider the multifactorial etiology of urinary incontinence. In the context of cervical cancer, a study demonstrated that women with gynecological cancer and vaginal dryness experienced a greater frequency of urinary incontinence compared to those without [35]. Therefore, there are additional factors contributing to the development of complications, aside from radiation therapy alone. This also applies to fistula, where additional clinical risk factors are well established [36]. In this study, compliance with GGE recommendations was associated with a reduction in the likelihood of acute bladder and rectum as well as long-term vaginal and dermal toxicities occurring. The presented results indicate that increasing D2cc to the rectum could heighten the odds of experiencing long-term and acute complications for the rectum besides an additional higher chance for vaginal and dermal long-term complications when D2cc to the rectum increases. Therefore, compliance with the Gyn GEC-ESTRO recommendations should be encouraged to mitigate toxicities in the organs at risk.

The evidence and effectiveness of the GGE recommendations, following the two EMBRACE studies, is already well described in recent publications [27,37,38]. Results from this study were compared to the EMBRACE-II planning aims, which are also reflected in the current ESGO/ESTRO/ESP guidelines [9]. The outcomes of the EMBRACE-I study were 68% (95% CI [65, 70]) for disease-free survival and 74% (95% CI [72, 77]) for overall survival at 5 years [8]. Considering that the dose volume coverage of the retrospectively added target volumes was relatively poor compared to the recommendations, previously shown oncologic outcomes of this study, though somewhat inferior for overall survival, are still very comparable, especially for PFS. The hypotheses of the EMBRACE-II study, which aim to surpass the outcomes of the EMBRACE-I study as a whole, include inter alia better local and systemic control as well as improved survival times [7]. This study suggests a slight trend that increasing deviations from GGE recommendations may lead to poorer local tumor control. Furthermore, patients who received treatments in accordance to the Point A recommendations experienced superior survival times and local tumor control. Therefore, adhering to the recommendations listed in the current ESGO/ESTRO/ESP guideline should be advocated [9]. 

The potential limitations of this study may contribute to the observed trend of poorer overall survival. The above-noted larger HR-CTV volumes in this study could indicate a selection bias, whereby particularly challenging patients are highlighted in a large center such as the University Hospital of Muenster. It was previously demonstrated that large tumors constitute an independent risk factor with regard to treatment failure [38]. In this context, we were also able to demonstrate, via statistical analysis, that larger volumes are associated with greater deviations from the GGE recommendations. The unequal distribution of FIGO stages provides further evidence to support the hypothesis of a potential selection bias. In the EMBRACE-I study, only 15.2% of cases were classified as FIGO stage III according to the 2009 classification, with stage IIB being the most common at 51.7% [8]. In contrast, the most prevalent stage in this study was stage IIIC according to the 2018 classification, which affected 50% of cases. According to the 2009 classification, this would have corresponded to stage IVa, which was present in only 2.5% of EMBRACE-I cases [5,8]. It is worth noting that in 2009, any local lymph node metastases were also classified as stage IVa, but this changed to stage IIIC in 2018 [5]. The only more advanced stage in EMBRACE-I, IVb with distant metastasis, was observed in 7.3% of cases, which also involved para-aortic metastasis in the 2009 classification [5,8]. 

Another point worth mentioning is that the COVID-19 pandemic coincided with this observation period. A study conducted in Germany demonstrated that during the pandemic, the detection of cancer was less effective and the overall number of cancer diagnoses decreased [39]. However, for cervical cancer in particular, the incidence increased during the pandemic, most likely due to the introduction of a new screening in 2020 and the young screening population [39]. This finding contrasts with the results from other countries, making it challenging to ascertain the impact of the pandemic on this study [40]. 

The small patient cohort (n = 48) and the retrospective nature of the dataset, which is consequently of limited quality, represent additional limitations of this study. Due to the small number of cases and events, it was not possible to fit multivariable models to adjust for possible interactions between the various influencing variables. The time dependence of long-term toxicities could not be modeled, and competing events, such as death, could not be statistically accounted for due to the small sample size. The start of survival and event times were defined after the end of therapy, as some influencing variables were measured during or at the end of therapy. The comparability of these endpoints with other studies may therefore not be given. Prospective randomized trials are required to confirm the results of this study.

## 5. Conclusions

Definitive radiochemotherapy for locally advanced cervical cancer in this retrospective monocenter study led to results that are both acceptable and comparable with those of other trials in terms of patients’ oncologic outcomes and OAR toxicities. Incomplete therapy and advanced tumor stage, including lymph node involvement, worsened treatment results. Adding MRI for the planning and administration of the associated brachytherapy should be preferred, as this may result in improved patients’ survival times. This aligns with the recommendations set forth by the Gyn GEC-ESTRO, whose implementation of constraints for target volume and OAR could lead to reduced acute bladder, rectum, and long-term vaginal and dermal toxicities. Compliance with Point A recommendations could potentially benefit survival times as well as local tumor control. Conversely, an increase in deviations from GGE could potentially worsen local tumor control. The implementation of the aforementioned guidelines could therefore further improve patients’ oncologic outcome. It is imperative that recent and upcoming adjustments to treatment recommendations and guidelines by the GGE, as possibly anticipated in the context of the EMBRACE-II study, are closely monitored in order to continue providing optimal care for patients.

## Figures and Tables

**Table 1:** Results from univariable logistic regression model for local tumor control.

Variable		CR 38 (79%)	No CR 10 (21%)	Odds Ratio (95% CI)	*p*-Value
Age at start of therapy	x + 1 vs. x years	49.5 (39.3–64.0)	61.0 (46.5–68.3)	0.96 (0.91, 1.01)	0.134
FIGO stage	I	7 (15%)	0	NE	NE
	II	10 (21%)	3 (6%)	1.11 (0.24, 5.23)	0.894
	III	21 (44%)	7 (15%)	Reference	
Lymph node involvement	No lymph node involvement	21 (44%)	3 (6%)	Reference	
	Pelvic lymph node metastasis	8 (17%)	3 (6%)	0.38 (0.06, 2.29)	0.292
	Para-aortic lymph node metastasis	9 (19%)	4 (8%)	0.32 (0.06, 1.74)	0.188
Complete therapy	Yes	27 (56%)	6 (13%)	Reference	
	No	11 (23%)	4 (8%)	0.61 (0.14, 2.60)	0.505
EBRT boost received	No	10 (21%)	4 (8%)	Reference	
	Yes	28 (58%)	6 (13%)	1.87 (0.44, 8.01)	0.401
MRI pre EBRT or for BT	No MRI	12 (25%)	2 (4%)	Reference	
	At least one MRI	26 (54%)	8 (17%)	0.54 (0.1, 2.95)	0.478
MRI for BT	No MRI	25 (52%)	7 (15%)	Reference	
	At least one MRI	13 (27%)	3 (6%)	1.21 (0.27, 5.49)	0.802
GTVres D98 [Gy]	x + 1 vs. x Gy	55.9 (51.3–61.3)	52.0 (48.3–56.8)	1.07 (0.96, 1.20)	0.205
IR-CTV D98 [Gy]	x + 1 vs. x Gy	44.1 (39.7–46.4)	42.0 (36.0–45.2)	1.04 (0.93, 1.17)	0.474
HR-CTV D90 [Gy]	x + 1 vs. x Gy	55.1 (52.8–58.9)	53.8 (49.0–57.9)	1.08 (0.94, 1.23)	0.275
HR-CTV D98 [Gy]	x + 1 vs. x Gy	49.9 (46.5–53.7)	47.5 (45.3–51.8)	1.05 (0.93, 1.19)	0.418
GTVres D98 deviation from GGE	x + 1 vs. x Gy	34.1 (28.7–38.7)	38.0 (33.3–41.7)	0.93 (0.84, 1.04)	0.205
IR-CTV D98 deviation from GGE	x + 1 vs. x Gy	15.9 (13.6–20.3)	18.0 (14.8–24.0)	0.96 (0.86, 1.08)	0.474
HR-CTV D90 deviation from GGE	x + 1 vs. x Gy	29.9 (26.1–32.2)	31.2 (27.1–36.0)	0.93 (0.81, 1.06)	0.275
HR-CTV D98 deviation from GGE	x + 1 vs. x Gy	25.1 (21.3–28.5)	27.5 (23.2–29.7)	0.95 (0.84, 1.07)	0.418
IR-CTV V150 [%]	x + 1 vs. x %	9.3 (7.5–11.2)	8.0 (4.4–9.8)	1.30 (0.98, 1.73)	0.073
IR-CTV V200 [%]	x + 1 vs. x %	4.3 (3.5–5.8)	3.6 (2.1–4.6)	1.77 (1.01, 3.09)	0.046
HR-CTV V150 [%]	x + 1 vs. x %	20.7 (15.9–24.8)	16.5 (9.8–19.6)	1.12 (0.99, 1.27)	0.063
HR-CTV V200 [%]	x + 1 vs. x %	10.2 (7.4–12.0)	7.8 (4.5–9.4)	1.22 (0.98, 1.50)	0.073
Point A GGE	Recommendations not met	16 (33%)	5 (10%)	Reference	
	Recommendations met	22 (46%)	5 (10%)	1.38 (0.34, 5.56)	0.655

Results are shown as median an interquartile range (IQR, 25% quantile, 75% quantile) or frequencies (n) and percentages (%) within each CR group. Odds ratios, corresponding 95% confidence limits, and two-sided *p*-values from Wald test are from univariable logistic regressions; odds ratio for x + 1 vs. x represents the change in odds per unit of a continuous variable. Abbreviations: NE, not estimable; EBRT, external beam radiotherapy; BT, brachytherapy; GTVres, residual gross tumor volume; IR-CTV, intermediate-risk clinical target volume; HR-CTV, high-risk clinical target volume; and GGE, Gyn GEC-ESTRO. All total doses were calculated as EQD2_(α/β=10)_ and recorded as Gy.

**Table 2:** Results of the Cox regressions for progression-free survival (PFS).

Variable		Events/Total 13/43	Hazard Ratio (95% CI)	*p*-Value
Age at start of therapy	x + 1 vs. x years	-	1.01 (0.97, 1.06)	0.551 **
FIGO stage	I	1/7	Reference	
	II	3/12	1.62 (0.17, 15.57)	0.511 *
	III	9/24	2.69 (0.34, 21.29)	
Lymph node involvement	No lymph node involvement	5/23	Reference	
	Pelvic lymph node metastasis	3/9	1.50 (0.36, 6.27)	0.192 *
	Para-aortic lymph node metastasis	5/11	3.04 (0.86, 10.76)	
Complete therapy	Yes	10/32	Reference	
	No	3/11	1.07 (0.29, 3.91)	0.921 *
EBRT boost received	No	3/12	Reference	
	Yes	10/31	1.43 (0.39, 5.20)	0.586 *
MRI pre EBRT or for BT	No MRI	6/13	Reference	
	At least one MRI	7/30	0.36 (0.12, 1.09)	0.058 *
MRI for BT	No MRI	8/27	Reference	
	At least one MRI	5/16	0.95 (0.31, 2.91)	0.922 *
GTVres D98 [Gy]	x + 1 vs. x Gy	-	0.99 (0.93, 1.06)	0.839 **
IR-CTV D98 [Gy]	x + 1 vs. x Gy	-	1.00 (0.91, 1.09)	0.907 **
HR-CTV D90 [Gy]	x + 1 vs. x Gy	-	1.00 (0.93, 1.08)	0.943 **
HR-CTV D98 [Gy]	x + 1 vs. x Gy	-	1.01 (0.93, 1.09)	0.880 **
GTVres D98 deviation from GGE	x + 1 vs. x Gy	-	1.01 (0.94, 1.08)	0.839 **
IR-CTV D98 deviation from GGE	x + 1 vs. x Gy	-	1.01 (0.92, 1.10)	0.907 **
HR-CTV D90 deviation from GGE	x + 1 vs. x Gy	-	1.00 (0.93, 1.07)	0.943 **
HR-CTV D98 deviation from GGE	x + 1 vs. x Gy	-	0.99 (0.92, 1.07)	0.880 **
Point A GGE	Recommendations not met	7/19	Reference	
	Recommendations met	6/24	0.62 (0.21, 1.86)	0.385 *

Five patients with early progression during treatment were excluded; two-sided *p*-values are from * log-rank test or ** Wald test from univariable Cox regression; hazard ratio for x + 1 vs. x represents the change in hazard per unit of a continuous variable. Abbreviations: EBRT, external beam radiotherapy; BT, brachytherapy; GTVres, residual gross tumor volume; IR-CTV, intermediate-risk clinical target volume; HR-CTV, high-risk clinical target volume; and GGE, Gyn GEC-ESTRO. All total doses were calculated as EQD2_(α/β=10)_ and recorded as Gy.

**Table 3:** Results of the Cox regressions for overall survival (OS).

Variable		Events/Total 14/48	Hazard Ratio (95% CI)	*p*-Value
Age at start of therapy	x + 1 vs. x years	-	1.05 (1.01, 1.09)	0.027 **
FIGO stage	I	1/7	Reference	
	II	4/13	1.94 (0.22, 17.47)	0.691 *
	III	9/28	2.36 (0.30, 18.73)	
Lymph node involvement	No lymph node involvement	6/24	Reference	
	Pelvic lymph node metastasis	4/11	1.3 (0.46, 5.81)	0.592 *
	Para-aortic lymph node metastasis	4/13	1.82 (0.50, 6.61)	
Complete therapy	Yes	7/33	Reference	
	No	7/15	3.27 (1.13, 9.42)	0.020 *
EBRT boost received	No	4/14	Reference	
	Yes	10/34	0.97 (0.30, 3.08)	0.954 *
MRI pre EBRT or for BT	No MRI	5/14	Reference	
	At least one MRI	9/34	0.54 (0.18, 1.62)	0.257 *
MRI for BT	No MRI	10/32	Reference	
	At least one MRI	4/16	0.63 (0.20, 2.03)	0.432 *
GTVres D98 [Gy]	x + 1 vs. x Gy	-	1.01 (0.95, 1.07)	0.833 **
IR-CTV D98 [Gy]	x + 1 vs. x Gy	-	1.04 (0.95, 1.14)	0.400 **
HR-CTV D90 [Gy]	x + 1 vs. x Gy	-	1.01 (0.95, 1.08)	0.678 **
HR-CTV D98 [Gy]	x + 1 vs. x Gy	-	1.02 (0.96, 1.10)	0.515 **
GTVres D98 deviation from GGE	x + 1 vs. x Gy	-	0.99 (0.93, 1.06)	0.833 **
IR-CTV D98 deviation from GGE	x + 1 vs. x Gy	-	0.96 (0.88, 1.05)	0.400 **
HR-CTV D90 deviation from GGE	x + 1 vs. x Gy	-	0.99 (0.93, 1.05)	0.678 **
HR-CTV D98 deviation from GGE	x + 1 vs. x Gy	-	0.98 (0.91, 1.05)	0.515 **
Point A GGE	Recommendations not met	7/21	Reference	
	Recommendations met	7/27	0.88 (0.31, 2.51)	0.806 *

No patients were excluded; two-sided *p*-values are from * log-rank test or ** Wald test from univariable Cox regression; hazard ratio for x + 1 vs. x represents the change in hazard per unit of a continuous variable. Abbreviations: EBRT, external beam radiotherapy; BT, brachytherapy; GTVres, residual gross tumor volume; IR-CTV, intermediate-risk clinical target volume; HR-CTV, high-risk clinical target volume; and GGE, Gyn GEC-ESTRO. All total doses were calculated as EQD2_(α/β=10)_ and recorded as Gy.

**Table 4:** Results of the Cox regressions for recurrence-free survival (RFS).

Variable		Events/Total 8/38	Hazard Ratio (95% CI)	*p*-Value
Age at start of therapy	x + 1 vs. x years	-	1.02 (0.97, 1.08)	0.428 **
FIGO stage	I	1/7	Reference	
	II	1/10	0.59 (0.04, 9.40)	0.427 *
	III	6/21	2.01 (0.24, 16.73)	
Lymph node involvement	No lymph node involvement	3/21	Reference	
	Pelvic lymph node metastasis	2/8	1.60 (0.27, 9.61)	0.162 *
	Para-aortic lymph node metastasis	3/9	4.38 (0.85, 22.61)	
Complete therapy	Yes	5/27	Reference	
	No	3/11	2.16 (0.51, 9.11)	0.284 *
EBRT boost received	No	1/10	Reference	
	Yes	7/28	3.07 (0.38, 24.99)	0.270 *
MRI pre EBRT or for BT	No MRI	5/12	Reference	
	At least one MRI	3/26	0.14 (0.03, 0.61)	0.003 *
MRI for BT	No MRI	6/25	Reference	
	At least one MRI	2/13	0.45 (0.09, 2.27)	0.322 *
GTVres D98 [Gy]	x + 1 vs. x Gy	-	1.03 (0.95, 1.10)	0.492 **
IR-CTV D98 [Gy]	x + 1 vs. x Gy	-	1.02 (0.91, 1.14)	0.723 **
HR-CTV D90 [Gy]	x + 1 vs. x Gy	-	1.03 (0.96,1.11)	0.409 **
HR-CTV D98 [Gy]	x + 1 vs. x Gy	-	1.03 (0.95, 1.12)	0.436 **
GTVres D98 deviation from GGE	x + 1 vs. x Gy	-	0.98 (0.91, 1.05)	0.492 **
IR-CTV D98 deviation from GGE	x + 1 vs. x Gy	-	0.98 (0.87, 1.10)	0.723 **
HR-CTV D90 deviation from GGE	x + 1 vs. x Gy	-	0.97 (0.90, 1.04)	0.409 **
HR-CTV D98 deviation from GGE	x + 1 vs. x Gy	-	0.97 (0.89, 1.05)	0.436 **
Point A GGE	Recommendations not met	4/16	Reference	
	Recommendations met	4/22	0.72 (0.18, 2.86)	0.631 *

10 patients who did not achieve a CR were excluded; two-sided *p*-values are from * log-rank test or ** Wald test from univariable Cox regression; hazard ratio for x + 1 vs. x represents the change in hazard per unit of a continuous variable. Abbreviations: EBRT, external beam radiotherapy; BT, brachytherapy; GTVres, residual gross tumor volume; IR-CTV, intermediate-risk clinical target volume; HR-CTV, high-risk clinical target volume; and GGE, Gyn GEC-ESTRO. All total doses were calculated as EQD2_(α/β=10)_ and recorded as Gy.

**Table 5:** Total doses of dose–volume parameters for different target volumes of all 48 patients.

Dose–Volume Parameter		GTVres	HR-CTV	IR-CTV
D98 [Gy] EQD2_(α/β=10)_	Median	55.3	49.6	43.4
	IQR	50.2–60.8	46.5–53.3	39.5–46.2
	Min–Max	40.2–82.1	36.7–73.5	27.9–56.9
D90 [Gy] EQD2_(α/β=10)_	Median		55.0	
	IQR		51.8–58.6	
	Min–Max		45.4–83.6	
V150 [%]	Median		19.2	8.5
	IQR		15.0–24.4	7.1–11.1
	Min–Max		6.0–71.4	4.0–27.2
V200 [%]	Median		9.8	4.3
	IQR		6.9–11.8	3.1–5.4
	Min–Max		0.4–44.7	0.8–14.7

Abbreviations: IQR, interquartile range; GTVres, residual gross tumor volume; HR-CTV, high-risk clinical target volume; IR-CTV, intermediate-risk clinical target volume; and Min–Max, minimum–maximum.

**Table 6:** Dose deviations from GGE recommendations for different target volumes of all 48 patients.

	GTVres D98 [Gy]	IR-CTV D98 [Gy]	HR-CTV D90 [Gy]	HR-CTV D98 [Gy]
Median	34.7	16.6	30.0	25.4
IQR	29.2–39.8	13.8–20.5	26.4–33.2	21.7–28.5
Min–Max	7.9–49.8	3.1–32.1	1.4–39.6	1.5–38.3

Abbreviations: IQR, interquartile range; GTVres, residual gross tumor volume; HR-CTV, high-risk clinical target volume; IR-CTV, intermediate-risk clinical target volume; and Min–Max, minimum–maximum.

**Table 7:** Complication frequency for bladder and rectum.

	Acute Complications ^1^		Long-Term Complications ^2^	
	Bladder	Rectum	Bladder	Rectum
No complications	29 (60%)	17 (35%)	20 (42%)	18 (38%)
Grade I	11 (30%)	20 (42%)	5 (10%)	8 (17%)
Grade II	1 (2%)	8 (17%)	7 (15%)	11 (23%)
Grade III	7 (15%)	3 (6%)	8 (17%)	1 (2%)
Grade IV	0	0	3 (6%)	5 (10%)

^1^ According to CTCAE v5.0; ^2^ according to LENT-SOMA scale, cumulative for a median follow-up of 58.0 months (IQR 84–28) months, long-term complications could be evaluated for only 43 of 48 patients due to missing retrospective data.

## Data Availability

Due to access restrictions, data is not available for sharing.
